# DNA methylation in genes of longevity-regulating pathways: association with obesity and metabolic complications

**DOI:** 10.18632/aging.101882

**Published:** 2019-03-29

**Authors:** Francisca Salas-Pérez, Omar Ramos-Lopez, María L. Mansego, Fermín I. Milagro, José L. Santos, José I. Riezu-Boj, J. Alfredo Martínez

**Affiliations:** 1 Department of Nutrition, Food Science and Physiology; Center for Nutrition Research, University of Navarra, Pamplona 31008, Spain; 2 Department of Bioinformatics, Making Genetics S.L, Pamplona 31002, Spain; 3 CIBERobn, Fisiopatología de la Obesidad y la Nutrición, Carlos III Health Institute, Madrid 28029, Spain; 4 IdiSNA, Navarra Institute for Health Research, Pamplona 31008, Spain; 5 Department of Nutrition, Diabetes and Metabolism, School of Medicine, Pontificia Universidad Católica de Chile, Santiago 8331150, Chile; 6 Institute IMDEA Food, Madrid 28049, Spain; * Equal contribution

**Keywords:** epigenetics, aging, metabolic syndrome, obesity, insulin resistance

## Abstract

Aging is the main risk factor for most chronic diseases. Epigenetic mechanisms, such as DNA methylation (DNAm) plays a pivotal role in the regulation of physiological responses that can vary along lifespan. The aim of this research was to analyze the association between leukocyte DNAm in genes involved in longevity and the occurrence of obesity and related metabolic alterations in an adult population. Subjects from the MENA cohort (n=474) were categorized according to age (<45 vs 45>) and the presence of metabolic alterations: increased waist circumference, hypercholesterolemia, insulin resistance, and metabolic syndrome. The methylation levels of 58 CpG sites located at genes involved in longevity-regulating pathways were strongly correlated (FDR-adjusted< 0.0001) with BMI. Fifteen of them were differentially methylated (p<0.05) between younger and older subjects that exhibited at least one metabolic alteration. Six of these CpG sites, located at *MTOR* (cg08862778), *ULK1* (cg07199894), *ADCY6* (cg11658986), *IGF1R* (cg01284192), *CREB5* (cg11301281), and *RELA* (cg08128650), were common to the metabolic traits, and *CREB5*, *RELA*, and *ULK1* were statistically associated with age. In summary, leukocyte DNAm levels of several CpG sites located at genes involved in longevity-regulating pathways were associated with obesity and metabolic syndrome traits, suggesting a role of DNAm in aging-related metabolic alterations.

## Introduction

Improvements in health care and nutrition, more efficient infrastructure and access to basic supplies have been increasing life expectancy worldwide, leading to a shift towards older populations (Methods for Life Expectancy and Healthy Life Expectancy, WHO (2014)). However, this extended lifespan is associated with an increase in the prevalence of age-related diseases [1]. On the other hand, obesity and its comorbidities have been reported to decrease longevity and accelerate aging. For example, a recent report has associated obesity with shorter longevity [2]; where normal-weight men lived on average about six years more than morbidly obese men, whereas morbidly obese women tended to live two years less than normal-weight women. Similarly, there is a relationship between obesity-related diseases and mortality or years of life lost (YLL). It has been estimated that obesity-related diseases increase lessened life years by 0.2 to 11.7 years depending on age BMI, gender and ethnic background [3].

Aging is an unavoidable physiological process, characterized by a progressive decline of functions in tissues and organs and is a risk factor for several pathological conditions including metabolic and cardiovascular diseases, neurodegenerative disorders and cancer [4,5]. In this context, aging is considered a major factor contributor to abdominal obesity, insulin resistance, type 2 diabetes and metabolic syndrome [6]. Interestingly, cases of extreme longevity exhibit a healthier phenotype associated to a lower prevalence of overweight and obesity, and lower blood pressure [7]. Mechanisms involved in the aging process are diverse and include genomic instability, telomere shortening, deregulated nutrient sensing, mitochondrial dysfunction, cellular senescence, stem cell exhaustion, altered cellular senescence, loss of proteostasis, and epigenetic changes [8,9]. Longevity regulating pathways encompass several genes and associated signaling pathways that can modulate processes such as autophagy, protein synthesis, nutrient sensing, mitochondrial function, oxidative stress, among others [10]. Some of the signaling pathways more intrinsically associated with longevity are those of the Insulin/Insulin Like Growth Factor (IGF-1) system, mammalian target of rapamycin (mTOR) and Sirtuin 1 (SIRT1). In invertebrate species, it has been demonstrated that a reduced signaling in insulin/IGF-1 can increase lifespan [11,12]. On the other hand, mTOR is a sensor that integrates environmental and intracellular signals. It has been shown that, the inhibition of mTORC1 with rapamycin increases lifespan in several animal models, which opens the door to new therapeutic approaches focused on aging [13]. Moreover, sirtuins also play a key role in longevity, where brain-specific Sirt1-overexpressing transgenic mice show significant life span extension, and aged mice exhibit phenotypes consistent with a delay in aging. SIRT1 can be modulated by caloric restriction, which extends lifespan in several organisms, and is the target of resveratrol, which has the ability to extend the lifespan of yeast, worms, and flies [14–16].

Altered epigenetic landscapes have been described in relation to aging. One of the most studied epigenetic mechanisms is DNA methylation, a dynamic process that controls genomic integrity and transcriptional activity. DNA methylation consists in the addition of a methyl group at the carbon 5 position of cytosine ring to obtain 5-methylcitosine [17], occurs at specific sites and can vary along cycles of life [18]. Changes in DNA methylation affects crucial processes such as chromatin states, gene expression, and cell renewal, among others [19]. In general terms, CpG methylation within promoters can lead to transcriptional repression and promoter regions from highly expressed genes are hypomethylated [20]. However, gene expression could be also affected by changes in DNA methylation in regions such as 5` UTR, 3` UTR or gene body, although the mechanism is less understood [20]. During aging, mammalian cells undergo global DNA hypomethylation, especially at repetitive transposable sequences, which mostly occurs in a stochastic manner, as well as local DNA hypermethylation [21]. Therefore, DNA hypomethylation appears to be a key factor associated to aging and longevity processes. Our group has previously reported that the methylation levels of 55 CpG sites in white blood cells were significantly associated with age [22]. In older subjects, global DNA methylation patterns have been correlated with frailty, which is related with the relaxation of the epigenetic control impacting in functional decline [23]. In contrast to germ-line inherited genetic mutations, DNA methylation is theoretically reversible due to the action environmental factors [24,25], which opens the door to modulate aging-related epigenetic marks through specific lifestyle and dietary interventions.

The use of epigenetic techniques and algorithms could predict the risk of a disease considering inter-individual epigenetic variability and be useful to prevent disease progression. The aim of this study is to analyze DNA methylation patterns of genes involved in longevity-regulating pathways in young and old subjects that exhibit metabolic alterations in order to understand the implication of these epigenetic DNA signatures in the development of aging-related metabolic complications.

## RESULTS

### Demographic, anthropometric and metabolic characteristics

**Figure 1:**
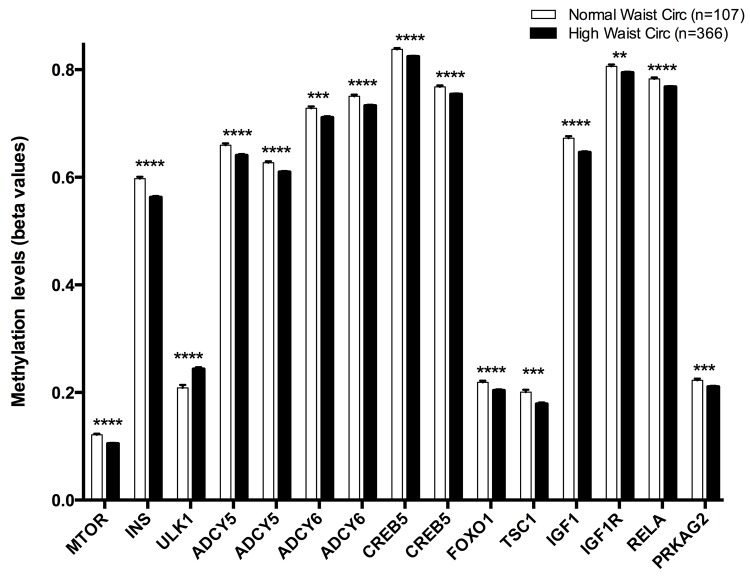
Methylation levels (beta values mean ± SEM) of CpGs located at genes of the longevity-regulating pathway in relation to waist circumference categories after age and sex adjustments. Normal waist circumference vs High waist circumference levels, p<0.01**; p<0.001***; p<0.0001****. Cut-off value between both groups was 102 cm for men and 88 cm for women.

Demographic, anthropometric and metabolic characteristics of the whole population ordered by median age are shown (Table 1). Population was divided according to median age (45 years) and as expected, older subjects (≥ 45 years) presented increased body mass index (BMI), waist circumference (WC), systolic and diastolic blood pressure (HT), insulin resistance (IR), and circulating cholesterol and triglyceride levels when compared to younger individuals. Differences in cholesterol levels were associated mainly to an increase in low-density lipoprotein cholesterol (LDL-c) and no significant differences were detected in high-density lipoprotein cholesterol (HDL-c) (Figure 1).

**Table 1:** Demographic, anthropometric and metabolic characteristics of the whole population and stratified by median age.

**Variable**		**All (n=474)**	**<45 years (n=224)**		**≥45 years (n=250)**
Age (years)	47.2±14.1			34.5±7.6	58.3±8.3^*^
Men/women	171/303			60/164	111/139^*^
Body weight (kg)	81.6±19.1			80.2±20.0	83.0±18.2
BMI (kg/m^2^)	30.1±5.6			29.0±6.0	30.9±5.2^*^
WC (cm)	95.7±16.1			91.4±17.6	99.7±13.4^*^
SBP (mm Hg)	121±28			101±35	124±42^*^
DBP (mm Hg)	89±25			75±32	90±32^*^
Glucose (mg/dl)	102±30			90±19	113±33^*^
Insulin (mIU/L)	9.6±7.0			9.0±6.4	10.7±7.8^*^
HOMA-IR index	2.44±2.28			2.13±1.98	2.96±2.64^*^
Total cholesterol (mg/dl)	205±40			191±37	216±39^*^
HDL-c (mg/dl)	53±13			54±14	54±13
LDL-c (mg/dl)	127±37			117±36	137±35^*^
Triglycerides (mg/dl)	120 ±72			105±70	132±73^*^

Categorical variables are presented as number of cases and continuous variables as means ± standard deviations. BMI: body mass index; WC: waist circumference; SBP: systolic blood pressure; DBP: diastolic blood pressure; HOMA-IR index: homeostatic model assessment-insulin resistance index; HDL-c: high-density lipoprotein cholesterol; LDL-c: low-density lipoprotein cholesterol. ^*^<45 years vs ≥45 years, p<0.05.

### DNA methylation in genes of longevity-regulating pathways in relation to BMI

Longevity and lifespan are influenced by genetics, the environment, and lifestyle. As phenotypes can vary substantially among individuals, we analyzed if DNA methylation of genes involved in longevity***-***regulating pathways is deregulated in presence of metabolic alterations. These genes were identified by using KEGG database (“Longevity-regulating pathway”). A first analysis was performed by correlating all DNA methylation sites across the 450K array with BMI. Among the 13,268 CpGs significantly associated with BMI (p<0.05), we focused on the 58 methylation sites that located in genes participating in the longevity-regulating pathway (FDR-adjusted p-value <0.0001) (Supplementary Table 2, Supplementary Table 1). Hereafter, 25 of these CpG sites were statistically significant (p<0.05) between younger (<45 y) and older subjects (≥45 y), and genomic and statistical data of these 25 CpG sites are shown (Tables 2-3). In a second step, we decided to perform a search of this 25 CpG sites in public repositories such as Gene Expression Omnibus (GEO) databases with metabolic phenotypes related to our study population (Supplementary Table 2). This analysis was conducted in four tissues such as PBMC (peripheral blood mononuclear cells), liver biopsies, subcutaneous and visceral fat to evaluate the methylation state in selected CpG sites (Table 4). GSE76399 compared insulin resistant and insulin sensitive individuals in PBMC, whereas GSE65057 compared obese and non-obese individuals in liver samples. In liver tissue from obese subjects, 13 CpG sites were hypomethylated showing a similar pattern in comparison to our results (blue lines in Table 4). In adipose tissue, we found only a hypermethylation for cg07199894 in *ULK1* gene in omental fat and hypomethylation for cg11322849 (*INS*), cg14844401 (*ADCY5*) and cg14323456 (*RHEB)* in subcutaneous fat, but it is important to note that sample size, gender and BMI were very different among studies. It is noteworthy that we found more coincidence between our results (that were based on the CpGs that were differential according to BMI) and the study comparing the methylation patterns between obese and non-obese subjects in liver, than to the study performed in PBMC (similar to our cells) but which compared insulin resistant versus insulin sensitive individuals. Another important difference between our study and the GSE76285 performed also in PBMC is that the population studied in GSE76285 was composed by subjects with extreme obesity (BMI >35), which makes difficult to find the same CpGs in both studies.

**Table 2:** Genomic data of the CpG sites located at longevity-regulating pathway genes that were statistically associated with BMI.

	**Illumina_ID**	**Gene name**	**Gene symbol**	**Chromosomal (CHR) position^2^**	**Genomic region**	**p-value**	**FDR adjusted** **p-value**	**Limma B**
**1**	cg08862778	Mechanistic target of rapamycin kinase	*MTOR*	1:11322643	TSS200	1.8E-13	3.3E-10	18.8
**2**	cg11322849	Insulin, transcript variant 1	*INS*	11:2182783	TSS1500	1.5E-10	5.1E-08	12.2
**3**	cg07199894	Unc-51 like autophagy activating kinase 1	*ULK1*	12:132379104	TSS200	1.3E-08	1.4E-06	7.83
**4**	cg11658986	Adenylate cyclase 6	*ADCY6*	12:49177605	1stExon	1.5E-08	1.5E-06	7.7
**5**	cg14862787	cAMP responsive element binding protein 5	*CREB5*	7:28507879	5'UTR	4.1E-08	3.1E-06	6.7
**6**	cg05792022	Forkhead box O1	*FOXO1*	13:41239732	1stExon	4.7E-08	3.5E-06	6.6
**7**	cg04149773	Adenylate cyclase 6	*ADCY6*	17:78518437	TSS200	1.4E-07	7.8E-06	5.5
**8**	cg14113970	Calcium/calmodulin dependent protein kinase IV, transcript variant 1	*CAMK4*	5:110789928	Body	2.1E-07	1.0E-05	5.1
**9**	cg14844401	Adenylate cyclase 5, transcript variant 1	*ADCY5*	3:123133741	Body	2.3E-07	1.1E-05	5.0
**10**	cg14267811	TSC complex subunit 1, transcript variant 4	*TSC1*	9:135819425	5'UTR	3.2E-07	1.4E-05	4.7
**11**	cg01284192	Insulin like growth factor 1 receptor, transcript variant 1	*IGF1R*	15:99500473	Body	4.1E-07	1.6E-05	4.5
**12**	cg24061580	Protein kinase AMP-activated non-catalytic subunit gamma 2, transcript variant a,	*PRKAG2*	7:151573966	5'UTR	4.7E-07	1.8E-05	4.4
**13**	cg06223834	Adenylate cyclase 9	*ADCY9*	16:4103161	Body	6.2E-07	2.2E-05	4.1
**14**	cg19503731	AKT serine/threonine kinase 3, transcript variant 2	*AKT3*	1:244007303	TSS1500	1.3E-06	3.8E-05	3.4
**15**	cg13154908	Phosphatidylinositol-4,5-bisphosphate 3-kinase catalytic subunit alpha	*PIK3CA*	3:178869001	5'UTR	1.5E-06	4.3E-05	3.2
**16**	cg18237616	Ras homolog, mTORC1 binding	*RHEB*	7:151191667	Body	2.0E-06	5.2E-05	3.0
**17**	cg14323456	Ras homolog, mTORC1 binding	*RHEB*	7:151205434	Body	2.0E-06	5.3E-05	2.9
**18**	cg01781374	Calcium/calmodulin dependent protein kinase IV, transcript variant 1	*CAMK4*	5:110777659	Body	2.1E-06	5.4E-05	2.9
**19**	cg11301281	cAMP responsive element binding protein 5, transcript variant 2	*CREB5*	7:28513286	5'UTR	2.4E-06	6.0E-05	2.8
**20**	cg02823066	Insulin like growth factor 1, transcript variant 3	*IGF1*	12:102819748	Body	2.5E-06	6.1E-05	2.7
**21**	cg20300093	Adenylate cyclase 5, transcript variant 1	*ADCY5*	3:123138250	Body	2.7E-06	6.6E-05	2.6
**22**	cg14077232	Euchromatic histone lysine methyltransferase 1, transcript variant 1	*EHMT1*	9:140656200	Body	2.7E-06	6.6E-05	2.6
**23**	cg08128650	RELA proto-oncogene, NF-kB subunit, transcript variant 1	*RELA*	11:65426704	Body	3.3E-06	7.6E-05	2.5
**24**	cg19418273	cAMP responsive element binding protein 3 like 2, transcript variant 1	*CREB3L2*	7:137562440	3'UTR	3.8E-06	8.3E-05	2.3
**25**	cg14072989	Ribosomal protein S6 kinase B1, transcript variant 1	*RPS6KB1*	17:57974225	Body	4.5E-06	9.3E-05	2.2

Data are sorted by FDR values. ^1^CpG identifier, ^2^CpG locations were mapped using GRCh37 version of the genome from Ensembl platform. BMI: body mass index; CHR: chromosome; FDR: False Discovery rate; B: LIMMA B-statistic from LIMMA.

**Table 3:** Methylation levels of CpG sites located at genes of the longevity-regulating pathways and differences between both age groups.

	**CpG**	**Gene**	**<45 years**	**≥ 45 years**	**p-value**
1	cg08862778	*MTOR*	0.111 ± 0.001	0.106 ± 0.001	0.0422
2	cg11322849	*INS*	0.576 ± 0.003	0.565 ± 0.003	0.0115
3	cg07199894	*ULK1*	0.227 ± 0.004	0.243 ± 0.003	0.0068
4	cg11658986	*ADCY6*	0.721 ± 0.002	0.709 ± 0,002	0.0016
5	cg14862787	*CREB5*	0.833 ± 0.001	0.822 ± 0.001	< 0.0001
6	cg05792022	*FOXO1*	0.213 ± 0.002	0.201 ± 0.002	0.0002
7	cg04149773	*ADCY6*	0.741 ± 0.002	0.732 ± 0.002	0.0019
8	cg14113970	*CAMK4*	0.774 ± 0.003	0.759 ± 0.004	0.0198
9	cg14844401	*ADCY5*	0.649 ± 0.002	0.641 ± 0.002	0.0343
10	cg14267811	*TSC1*	0.190 ± 0.003	0.177 ± 0.003	0.0049
11	cg01284192	*IGF1R*	0.801 ± 0.002	0.792 ± 0.002	0.0051
12	cg24061580	*PRKAG2*	0.217 ± 0.002	0.209 ± 0.001	0.0067
13	cg06223834	*ADCY9*	0.773 ± 0.003	0.758 ± 0.003	0.0009
14	cg19503731	*AKT3*	0.865 ± 0.001	0.860 ± 0.001	0.0401
15	cg13154908	*PIK3CA*	0.798 ± 0.003	0.788 ± 0.003	0.0497
16	cg18237616	*RHEB*	0.840 ± 0.002	0.830 ± 0.002	0.0039
17	cg14323456	*RHEB*	0.821 ± 0.003	0.810 ± 0.004	0.0456
18	cg01781374	*CAMK4*	0.831 ± 0.002	0.822 ± 0.002	0.0099
19	cg11301281	*CREB5*	0.763 ± 0.001	0.752 ± 0.001	< 0.0001
20	cg02823066	*IGF1*	0.658 ± 0.002	0.647 ± 0.002	0.0028
21	cg20300093	*ADCY5*	0.618 ± 0.002	0.609 ± 0.002	0.0032
22	cg14077232	*EHMT1*	0.823 ± 0.002	0.812 ± 0.002	0.0026
23	cg08128650	*RELA*	0.777 ± 0.001	0.766 ± 0.002	0.0001
24	cg19418273	*CREB3L2*	0.844 ± 0.002	0.835 ± 0.003	0.0316
25	cg14072989	*RPS6KB1*	0.825 ± 0.002	0.814 ± 0.003	0.0308

CpG beta values are grouped by age and p values are shown. Data are expressed as beta values mean ± SEM.

**Table 4:** Methylation levels of 25 CpG sites located at genes of the longevity-regulating pathways that were differentially methylated between younger (<45 y) and older subjects (≥45 y) in GEO datasets. GSE76399 compared insulin resistant and insulin sensitive individuals in PBMC, whereas GSE65057 compared obese and non-obese individuals in liver samples.

		**PMBC (GSE76399)**			**Liver tissue (GSE65057)**		
**CpG**	**Gene**	**Ins- Sens (n=40)**	**Ins- Resist (n=40)**	**P value**	**Non-Obese (n=7)**	**Obese (n=8)**	**P value**
cg08862778	*MTOR*	0.022 ± 0.001	0.024 ± 0.001	0.145	0.069 ± 0.004	0.079 ± 0.009	0.357
cg11322849	*INS*	0.692 ± 0.006	0.668 ± 0.016	0.154	0.676 ± 0.008	0.626 ± 0.017	0.025
cg07199894	*ULK1*	0.199 ± 0.006	0.206 ± 0.007	0.447	0.319 ± 0.011	0.311 ± 0.014	0.661
cg11658986	*ADCY6*	0.675 ± 0.008	0.677 ± 0.007	0.893	0.621 ± 0.010	0.566 ± 0.010	0.003
cg14862787	*CREB5*	0.875 ± 0.003	0.856 ± 0.016	0.255	0.719 ± 0.011	0.720 ± 0.020	0.968
cg05792022	*FOXO1*	0.209 ± 0.004	0.204 ± 0.005	0.470	0.261 ± 0.007	0.237 ± 0.014	0.170
cg04149773	*ADCY6*	0.724 ± 0.018	0.726 ± 0.019	0.959	0.797 ± 0.006	0.748 ± 0.019	0.035
cg14113970	*CAMK4*	0.881 ± 0.027	0.827 ± 0.037	0.236	0.850 ± 0.008	0.795 ± 0.026	0.078
cg14844401	*ADCY5*	0.698 ± 0.010	0.658 ± 0.028	0.186	0.657 ± 0.011	0.631 ± 0.016	0.202
cg14267811	*TSC1*	0.209 ± 0.005	0.211 ± 0.005	0.775	0.219 ± 0.006	0.212 ± 0.012	0.605
cg01284192	*IGF1R*	0.842 ± 0.005	0.837 ± 0.005	0.471	0.815 ± 0.009	0.774 ± 0.009	0.008
cg24061580	*PRKAG2*	0.199 ± 0.004	0.186 ± 0.005	0.060	0.247 ± 0.004	0.244 ± 0.008	0.760
cg06223834	*ADCY9*	0.867 ± 0.006	0.821 ± 0.030	0.143	0.838 ± 0.010	0.795 ± 0.015	0.042
cg19503731	*AKT3*	0.953 ± 0.003	0.946 ± 0.003	0.086	0.902 ± 0.006	0.895 ± 0.010	0.578
cg13154908	*PIK3CA*	0.955 ± 0.003	0.949 ± 0.003	0.087	0.927 ± 0.004	0.909 ± 0.006	0.029
cg18237616	*RHEB*	0.919 ± 0.018	0.914 ± 0.018	0.860	0.712 ± 0.017	0.717 ± 0.015	0.819
cg14323456	*RHEB*	0.956 ± 0.004	0.956 ± 0.003	0.958	0.929 ± 0.010	0.886 ± 0.015	0.039
cg01781374	*CAMK4*	0.926 ± 0.016	0.878 ± 0.023	0.097	0.839 ± 0.012	0.852 ± 0.012	0.457
cg11301281	*CREB5*	0.768 ± 0.019	0.733 ± 0.024	0.256	0.636 ± 0.011	0.584 ± 0.016	0.024
cg02823066	*IGF1*	0.649 ± 0.021	0.619 ± 0.037	0.481	0.319 ± 0.015	0.323 ± 0.013	0.830
cg20300093	*ADCY5*	0.639 ± 0.017	0.594 ± 0.028	0.172	0.708 ± 0.009	0.647 ± 0.027	0.068
cg14077232	*EHMT1*	0.954 ± 0.002	0.956 ± 0.002	0.479	0.905 ± 0.005	0.901 ± 0.013	0.806
cg08128650	*RELA*	0.799 ± 0.011	0.768 ± 0.017	0.138	0.698 ± 0.006	0.666 ± 0.015	0.077
cg19418273	*CREB3L2*	0.964 ± 0.003	0.960 ± 0.004	0.527	0.940 ± 0.005	0.907 ± 0.007	0.003
cg14072989	*RPS6KB1*	0.948 ± 0.006	0.947 ± 0.006	0.994	0.904 ± 0.013	0.839 ± 0.023	0.033

Ins-Sens: Insulin sensitive; Ins-Resist: Insulin Resistant. PBMC: Peripheral Blood Mononuclear Cells. Blue boxes represent significant changes in DNAm (p<0.05).

### Methylation levels in longevity-regulating pathways and metabolic alterations

From those 25 CpG sites that were differentially methylated among age categories, a second filter was applied. Subjects were categorized according the presence of metabolic alterations and the new selection was made based in those CpGs that were either hypo- or hypermethylated according to age and metabolic traits, in the same direction: those CpGs that were hypomethylated in the disease must be also hypomethylated with age, and vice versa. To perform this analysis, binary categories were created (absence or presence of the condition) as previously described: Abdominal obesity, hypercholesterolemia, insulin resistance and metabolic syndrome. Fifteen CpG sites were positively associated with abdominal obesity: cg08862778 (*MTOR*), cg11322849 (*INS*), cg07199894 (*ULK1*), cg14844401 (*ADCY5*), cg20300093 (*ADCY5*), cg11658986 (*ADCY6*), cg04149773 (*ADCY6*), cg14862787 (*CREB5*), cg11301281 (*CREB5*), cg05792022 (*FOXO1*), cg14267811 (*TSC1*), cg02823066 (*IGF1*), cg01284192 (*IGF1R*), cg08128650 (*RELA*), cg24061580 (*PRKAG2*) (Figure 1). Of these 15 CpG sites, cg07199894 (*ULK1*) was the only hypermethylated.

**Figure 2:**
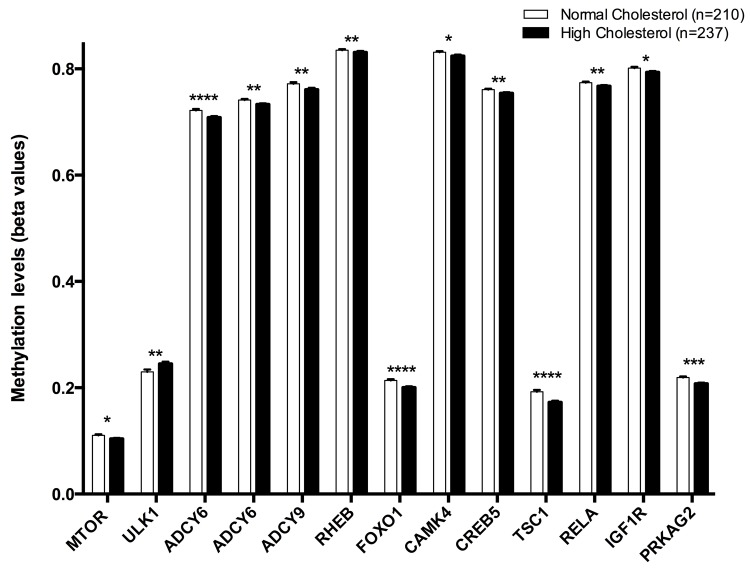
Methylation levels (beta values mean ± SEM) of CPGs located at genes of the longevity-regulating pathway in relation to total cholesterol categories after age and sex adjustments. Normal cholesterol vs High cholesterol levels, p<0.05*; p<0.01**; p<0.001***; p<0.0001****. Cut-off value between both groups was 200 mg/dl of total cholesterol in plasma.

Thirteen CpG were positively associated with hypercholesterolemia: cg08862778 (*MTOR*), cg07199894 (*ULK1*), cg11658986 (*ADCY6*), cg04149773 (*ADCY6*), cg06223834 (*ADCY9*), cg18237616 (*RHEB*), cg05792022 (*FOXO1*), cg01781374 (*CAMK4*), cg11301281 (*CREB5*), cg14267811 (*TSC1*), cg08128650 (*RELA*), cg01284192 (*IGF1R*), cg24061580 (*PRKAG2*) (Figure 2).

**Figure 3:**
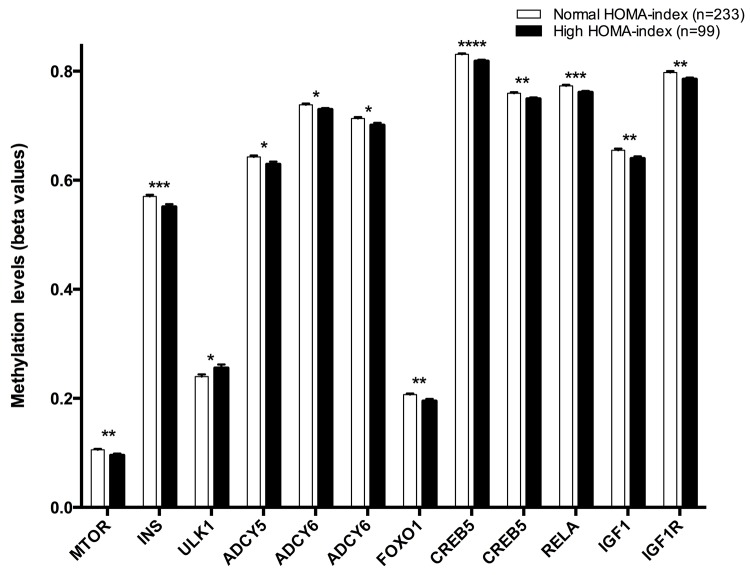
Methylation levels (beta values mean ± SEM) of CPGs located at genes of the longevity-regulating pathway in relation to HOMA-index categories after age and sex adjustments. Normal HOMA-index vs High HOMA-index levels, p<0.05*; p<0.01**; p<0.001***; p<0.0001****. Cut-off value between both groups was a 2.4 HOMA-IR index, higher levels was considered insulin resistant.

Twelve CpG sites were associated with HOMA index: cg08862778 (*MTOR*), cg11322849 (*INS*), cg07199894 (*ULK1*), cg11658986- cg04149773 (*ADCY6*), cg14862787-cg11301281 (*CREB5*), cg05792022 (*FOXO1*), cg14844401 (*ADCY5*), cg01284192 (*IGF1R*), cg02823066 (*IGF1*), cg08128650 (*RELA*) (Figure 3).

**Figure 4:**
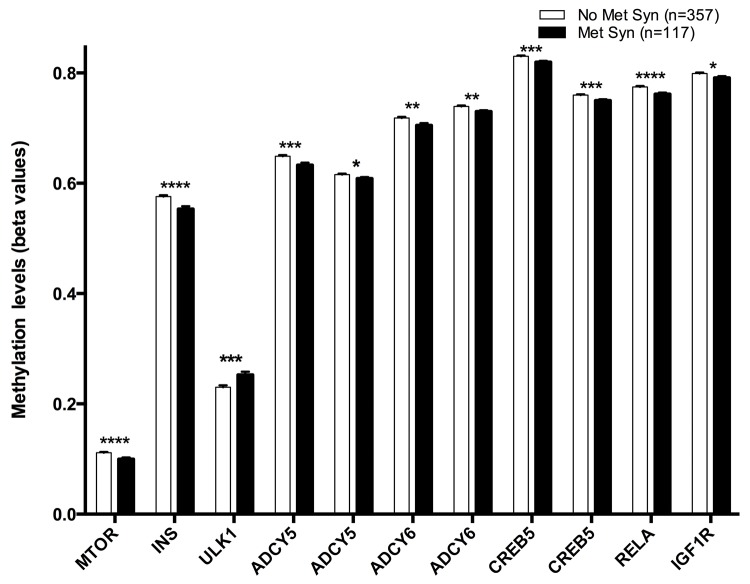
Methylation levels (beta values mean ± SEM) of CPGs located at genes of the longevity-regulating pathway in relation to Metabolic Syndrome categories after age and sex adjustments. Non-Metabolic syndrome vs Metabolic syndrome, p<0.05*; p<0.01**; p<0.001***; p<0.0001****. Metabolic syndrome was defined as the presence of three of five criteria: large waist circumference reduced HDL-c, hypertriglyceridemia, hypertension and fasting hyperglycemia.

Finally, eleven CpG sites were associated with metabolic syndrome: cg08862778 (*MTOR*), cg11322849 (*INS*), cg07199894 (*ULK1*), cg11658986-cg04149773 (*ADCY6*), cg14862787-11301281 (*CREB5*), cg14844401-cg20300093 (*ADCY5*), cg01284192 (*IGF1R*) and cg08128650 (*RELA*) (Figure 4).

In an attempt to define age-specific CpG sites, we decided to perform a characterization in the older subjects according to their metabolic phenotype. Starting from the 58 CpG sites associated with BMI residing in longevity regulating genes in the whole population (FDR<0.0001), and using the same criteria to split the population based on metabolic parameters as previously described, we observed that new CpG sites (blue lines in Table 5) appear differentially methylated in hyperglycemia, insulin resistance and hypertriglyceridemia but not in hypercholesterolemia. Interestingly, 8 CpGs (cg11322849, cg07199894, cg14862787, cgcg14844401, cg01091261, cg06223834, cg08128650, cg04149773 in gray lines, Table 5) coincide when the whole population and the old sub-population are analyzed, which suggests that these genes could have a greater impact in metabolic regulation (Table 5). Moreover, *PRKAG2* (cg20406576) and *EHMT2* (cg00210002) are hypomethylated in subjects with hyperglycemia, HOMA-IR ≥2.5 and hypertriglyceridemia. In the case of *IRS1* (cg21511036), *AKT1S1* (cg03813033), *ADCY2* (cg12566890 and *EHMT2* (cg00210002) are hypomethylated, whereas *AKT1* (cg01749142), *FOXO3* (15283498), are hypermethylated in subjects with insulin resistance and hypertriglyceridemia.

**Table 5:** Methylation levels of CpG sites associated with BMI in the whole population and located at genes of the longevity-regulating pathways, according to metabolic phenotype in the older sub-population (≥ 45 years).

**CpG site**	**Gene**			**Normoglycemia (n=112)**	**Hyperglycemia (n=127)**			**P value**	
cg20406576	*PRKAG2*		0.802 ± 0.003		0.789 ± 0.003			0.005	
cg00210002	*EHMT2*		0.150 ± 0.004		0.136 ± 0.004			0.009	
**CpG site**	**Gene**		**HOMA-IR < 2.5 (n=75)**		**HOMA-IR >= 2.5 (n=53)**			**P value**	
cg21511036	*IRS1*		0.129 ± 0.002		0.119 ± 0.003			0.014	
cg20406576	*PRKAG2*		0.791 ± 0.003		0.776 ± 0.005			0.023	
cg01749142	*AKT1*		0.140 ± 0.004		0.158 ± 0.005			0.005	
cg15283498	*FOXO3*		0.799 ± 0.006		0.825 ± 0.004			0.003	
cg17848496	*IRS1*		0.818 ± 0.004		0.832 ± 0.003			0.037	
cg03813033	*AKT1S1*		0.111 ± 0.003		0.093 ± 0.002			0.000	
cg07012178	*PRKAG2*		0.755 ± 0.003		0.737 ± 0.003			0.001	
cg18028483	*SESN1*		0.751 ± 0.006		0.772 ± 0.006			0.033	
cg12566890	*ADCY2*		0.715 ± 0.003		0.702 ± 0.003			0.005	
cg13574337	*ADCY9*		0.665 ± 0.006		0.638 ± 0.005			0.003	
cg13796676	*SIRT1*		0.813 ± 0.006		0.834 ± 0.005			0.012	
cg06772578	*PPARGC1A*		0.783 ± 0.006		0.805 ± 0.005			0.015	
cg10421188	*CREB5*		0.637 ± 0.004		0.614 ± 0.004			0.001	
cg24937356	*PRKAA2*		0.792 ± 0.006		0.814 ± 0.004			0.013	
cg04932465	*CAMKK2*		0.666 ± 0.004		0.647 ± 0.005			0.006	
cg08315825	*IGF1R*		0.795 ± 0.007		0.819 ± 0.006			0.018	
cg00210002	*EHMT2*		0.136 ± 0.004		0.114 ± 0.004			0.001	
cg11322849	*INS*		0.557 ± 0.005		0.539 ± 0.006			0.044	
cg07199894	*ULK1*		0.254 ± 0.007		0.284 ± 0.006			0.006	
cg14862787	*CREB5*		0.819 ± 0.002		0.810 ± 0.003			0.042	
cg14844401	*ADCY5*		0.631 ± 0.005		0.614 ± 0.005			0.025	
cg01091261	*ADCY9*		0.799 ± 0.003		0.788 ± 0.004			0.048	
cg06223834	*ADCY9*		0.770 ± 0.004		0.786 ± 0.005			0.018	
cg08128650	*RELA*		0.762 ± 0.003		0.751 ± 0.003			0.042	
**CpG site**		**Gene**	**Non-Hypertriglyceridemia**			**Hypertriglyceridemia**	**P value**	
			**(n=177)**			**(n=55)**		
cg21511036		*IRS1*	0.138 ± 0.001			0.130 ± 0.003	0.030	
cg20406576		*PRKAG2*	0.798 ± 0.002			0.786 ± 0.004	0.025	
cg01749142		*AKT1*	0.127 ± 0.002			0.139 ± 0.004	0.014	
cg15283498		*FOXO3*	0.783 ± 0.004			0.805 ± 0.006	0.013	
cg03813033		*AKT1S1*	0.122 ± 0.002			0.110 ± 0.004	0.027	
cg12566890		*ADCY2*	0.721 ± 0.002			0.710 ± 0.004	0.019	
cg13613346		*EHMT2*	0.865 ± 0.004			0.881 ± 0.003	0.032	
cg00210002		*EHMT2*	0.149 ± 0.003			0.127 ± 0.004	0.000	
cg11322849		*INS*	0.568 ± 0.003			0.550 ± 0.006	0.010	
cg04149773		*ADCY6*	0.734 ± 0.002			0.725 ± 0.003	0.044	

In a second step, PathDIP was used to perform a pathway enrichment analysis and address the impact of these epigenetic marks on longevity-related molecular processes. This analysis showed that those CpG sites associated with the occurrence of metabolic alterations contributed significantly to the regulation of longevity-regulating pathways (Table 6). Moreover, multiple linear regressions adjusted by LARS (least-angle regression) were performed to determine which CpG sites contributed in a significant way to the metabolic traits. The resulting models show that the identified CpG sites have an important impact in abdominal obesity measured as waist circumference (adjusted r^2^:0.341), and also in insulin resistance expressed as HOMA-index (adjusted r^2^:0.153) (Table 6).

**Table 6:** Pathway enrichment analysis results ('q-value (FDR: BH-method) less than 0.05').

**Nº CpG**	**Pathway Source**	**Pathway Name**	**P value**	**Category**	**Genes**	**r^2^ Adj**
15	KEGG	Longevity regulating pathway	2.27E-26	All	*ADCY5, ADCY6, ADCY9, CAMK4, CREB5, FOXO1, IGF1, IGF1R, INS, MTOR, PRKAG2, RELA, RHEB, TSC1, ULK1*	0
12	KEGG	Longevity regulating pathway	3.23E-21	Cholesterol	*ADCY6, ADCY9, CAMK4, CREB5, FOXO1, IGF1R, MTOR, PRKAG2, RELA, RHEB, TSC1, ULK1*	0.072
10	KEGG	Longevity regulating pathway	8.67E-18	HOMA-Index	*ADCY5, ADCY6, CREB5, FOXO1, IGF1, IGF1R, INS, MTOR, RELA,ULK1*	0.153
12	KEGG	Longevity regulating pathway	3.23E-21	Waist Circumference	*ADCY5, ADCY6, CREB5, FOXO1, IGF1, IGF1R, INS, MTOR, PRKAG2, RELA, TSC1, ULK1*	0.341
8	KEGG	Longevity regulating pathway	2.30E-14	Metabolic Syndrome	*ADCY5, ADCY6, CREB5, IGF1R, INS, MTOR, RELA, ULK1*	0.092

KEGG: Kyoto Encyclopedia of Genes and Genomes. HOMA: homeostatic model assessment BMI: Body mass index.

**Figure 5:**
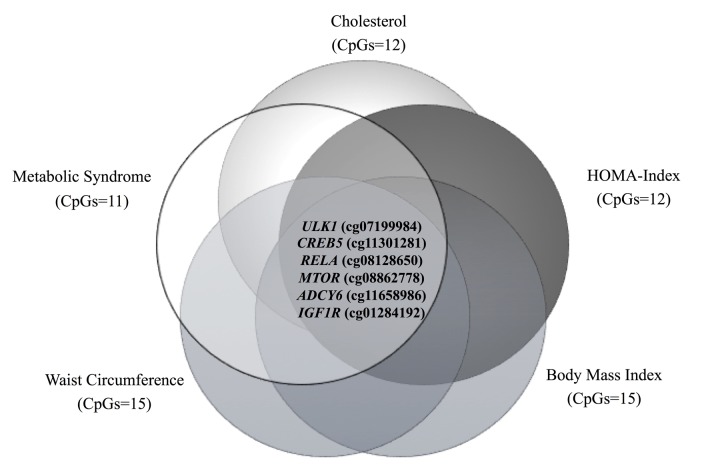
Venn diagram showing the common CpG sites differentially methylated between the different metabolic disturbances.

**Figure 6:**
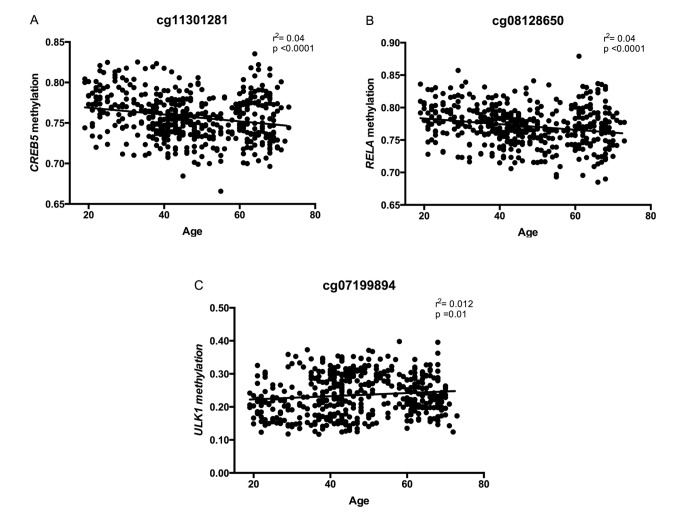
Correlations between DNA methylation levels (beta values) at CpGs located at genes of the longevity-regulating pathway and age, after sex adjustment. In (**A**) cg08128650, *RELA*, (**B**) cg11301281, *CREB5,* and (**C**) cg07199894, *ULK1* (n=474 subjects).

**Figure 7:**
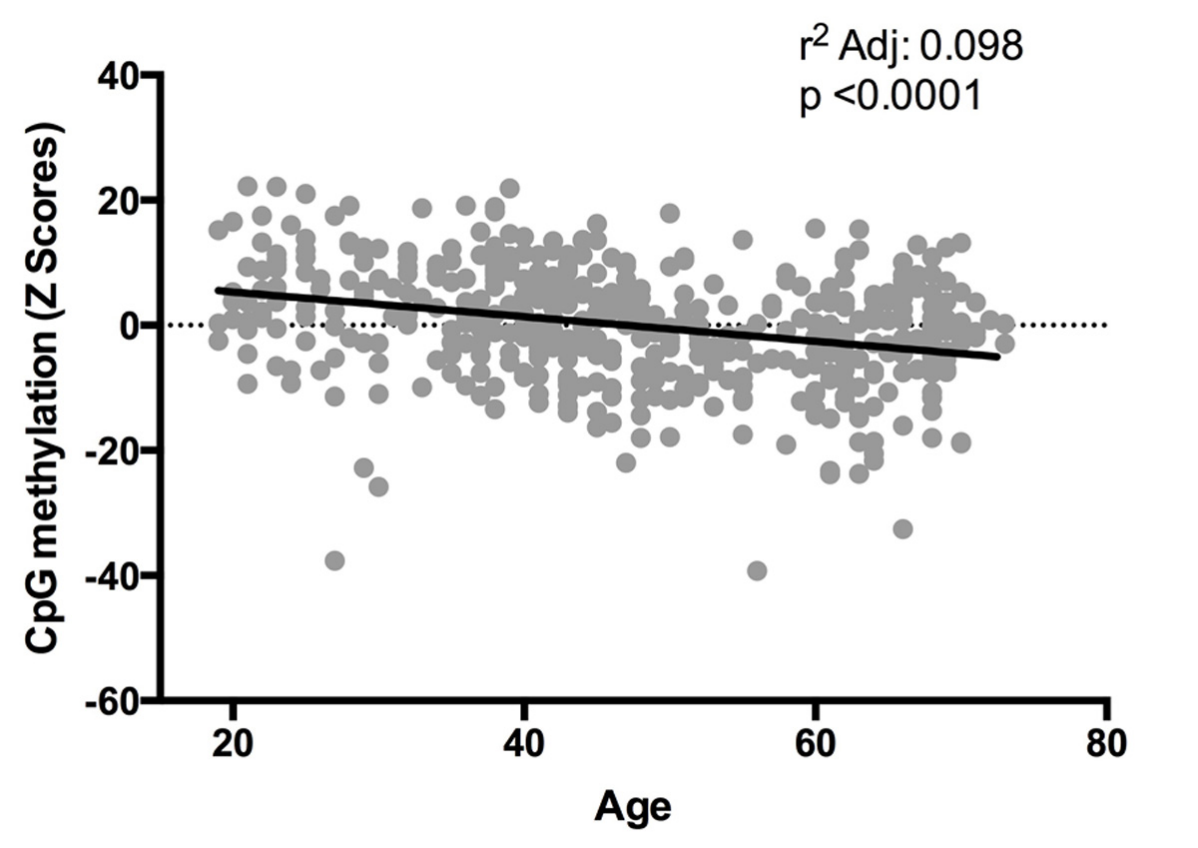
Relation between Z-scores of methylation levels and age. Values reflect the significant change in Z-scores from CpG methylation (25 CpG sites, n=474) according age, after BMI adjustment.

As mentioned before, a total of 15 CpG sites are hypo- or hypermethylated in older subjects, but only six of these CpG sites are common for all the metabolic alterations previously described (Figure 5). In a second approach, correlations between methylation levels and age were made for the six CpG sites located at the genes *MTOR*, *ULK1*, *ADCY6*, *IGFR1*, *CREB5* and *RELA*. In the case of cg08128650 (*RELA*) and cg11301281 (*CREB5*), methylation levels negative correlated with age, while cg07199984 (*ULK1*) showed a significant positive correlation (Figure 6). Considering that a small number of common CpG sites are significant associated with age and r^2^ adjusted values are small, we decided to create a z-score that includes those 25 CpG sites differentially methylated according to age previously described (Table 3) and a linear regression was performed analyzing this z-score with age, adjusted by BMI (Figure 7). This analysis showed a statistically significant association with age (r^2^:0.098, p<0.0001), where older subjects presented hypomethylation in CpG sites related to genes involved in longevity-regulating pathways.

### Analysis of DNA methylation in the older subpopulation (≥45 y)

DNA methylation patterns were analyzed using the whole population to define CpG sites related to BMI, but it is plausible to think that using a mixed population could mask a specific pattern related to age and whether BMI-associated CpGs differ significantly in young and older individuals. To address this, starting from normalized beta values, a new analysis was performed to identify CpG sites associated with BMI in young and old subjects, separately. Using the same criteria as described above (p-value <0.05), a total of 470 CpG sites were identified in <45 y subjects (n=220) while in ≥45 y subjects, (n=250) a total of 52,991 CpG sites. In a second step, we selected CpGs residing in longevity genes with a FDR value (p<0.0001) and this only could be possible in the older group where a total of 14 CpG sites were identified (Table 7). When the old sub-population is compared with the whole population, 6 CpGs (FDR<0.0001) are coincident in these two groups (cg14113970, cg13551841, cg15283498, cg21511036, cg06772578, cg07012178, in gray lines, Table 7).

**Table 7:** CpG sites residing in longevity genes identified in older subjects (FDR_≥ 45 y) in comparison to the whole population (FDR_Total).

**ID**	**Gene**	**FDR_≥45**	**FDR_Total**
cg04566392	*ADIPOR1*	1.89E-05	NA
cg08779982	*TSC2*	4.31E-05	NA
cg24773542	*EHMT2*	3.43E-05	NA
cg13804196	*PRKACG*	1.91E-05	NA
cg08048831	*IGF1R*	2.93E-05	1.00E-04
cg03310087	*IGF1R*	9.18E-05	5.00E-04
cg07340599	*CREB5*	8.48E-05	1.00E-04
cg21184115	*FOXO3*	1.29E-05	2.00E-04
cg14113970	*CAMK4*	8.66E-05	1.00E-05
cg13551841	*EHMT1*	4.79E-05	5.12E-07
cg15283498	*FOXO3*	2.76E-05	2.01E-06
cg21511036	*IRS1*	2.72E-06	5.01E-11
cg06772578	*PPARGC1A*	3.35E-05	3.83E-05
cg07012178	*PRKAG2*	1.35E-05	6.28E-05

FDR: False Discovery Rate

Four of these CpGs that were found in the older population (blue and green lines) had not appeared before (cg04566392, cg08779982, cg24773542, cg13804196) but two of them belong to the genes *EHMT2* and *PRKACG*, previously described in the whole population. Finally, four of these CpGs found in the older population also became significant (cg08048831, cg03310087, cg07340599, cg21184115, in purple lines, Table 7). These four CpGs in the whole population did not reach statistical significance, maybe because we are mixing both young and older populations, and when the older sub-population is analyzed alone, this effect disappears.

In a similar manner to that performed in the whole population, in the pursuit to obtain CpGs associated to metabolic disturbances in the sub-old population, we decided to split the old population (≥45 y) according to their metabolic phenotype and analyze these CpGs. In the old sub-population, from the 14 CpG sites that were associated with BMI in Table 7, we observed that three CpG sites were hypomethylated in hyperglycemia (cg13551841, cg08779982, cg21511036), two CpG sites were hypomethylated (cg13551841 and cg21511036) and one CpG residing in *ADIPOR1* (cg04566392) was hypermethylated in insulin resistance, and the CpG residing in *IRS1* (cg21511036) was hypomethylated in subjects with hypertriglyceridemia (Table 8). In this analysis, cg21511036 *(IRS1)* was hypomethylated in subjects with hyperglycemia, insulin resistance and hypertriglyceridemia. Those CpG sites were not previously described (blue lines). It is noteworthy that cg21511036 (*IRS1)* appears as a conserved hypomethylated CpG in the whole population and also, in the old sub-population (Tables 5 and 8), which suggests an important role in lipid and glucose disturbances.

**Table 8:** DNA methylation of CpG sites associated with BMI (FDR<0.0001) and residing in longevity regulating pathways according to metabolic phenotype in ≥ 45 y.

**CpG site**	**Gene**	**Normoglycemia**	**Hyperglycemia**	**P value**
		**(n=112)**	**(n=127)**	
cg13551841	*EHMT1*	0.708 ± 0.004	0.695 ± 0.004	0.023
cg08779982	*TSC2*	0.856 ± 0.002	0.848 ± 0.002	0.013
cg21511036	*IRS1*	0.137 ± 0.002	0.129 ± 0.002	0.013
				
**CpG site**	**Gene**	**HOMA-IR < 2.5**	**HOMA-IR >= 2.5**	**P value**
		**(n=75)**	**(n=53)**	
cg04566392	*ADIPOR1*	0.067 ± 0.002	0.076 ± 0.002	0.003
cg13551841	*EHMT1*	0.717 ± 0.004	0.690 ± 0.006	0.000
cg21511036	*IRS1*	0.146 ± 0.002	0.135 ± 0.003	0.002
				
**CpG site**	**Gene**	**Non-Hypertriglyceridemia**	**Hypertriglyceridemia**	**P value**
		**(n=177)**	**(n=55)**	
cg21511036	*IRS1*	0.135 ± 0.002	0.124 ± 0.003	0.004

We were not able to validate these results with other techniques due to the lack of DNA, but in an attempt to contrast these results, we decided to search these identified CpGs in GEO database. We did not find significant differences in CpG sites in selected datasets (Supplementary Table 2) in tissues as blood or visceral fat. In liver biopsies, two CpG sites (cg15283498-*FOXO3*; cg07012178-*PRKAG2*) were hypomethylated, whereas in subcutaneous adipose tissue from subjects with or without metabolic syndrome, cg07340599 (*CREB5)* and cg08779982 (*TSC2*) were hypomethylated.

## DISCUSSION

Longevity is considered the survival up to advanced ages [26]. Long-lived people are those who exceeds ≥90 years and in some cases, are individuals who have stabilized or avoided age-related diseases [27]. On the other hand, aging is an unavoidable process, characterized by a progressive decline of functions in tissues and organs and is associated to mortality [28]. Up to date, several GWAS and linkage and candidate gene association studies have identified genetic variants, such as those for apoliprotein E (*APOE*) and forkhead box O3 (*FOXO3A*), that have been consistently associated with longevity [29]. In the case of APOE, APOE ε2 isoform decrease the risk of cardiovascular disease and APOE ε4 isoform limits longevity [30] and FOXO3 is linked to insulin/insulin-like growth factor 1 (IGF1) signaling [31]. Another classic genetic model of increased lifespan in mammals is the growth hormone receptor (GHR) knock-out mouse, as well as its corresponding genetic defect in humans (Laron syndrome), which is characterized by extreme insulin sensitivity and protection to cancer [32,33]. These studies suggest that genetic variants are important contributors to the variability in longevity, but it is important to find other regulators of the longevity process. In this context, epigenetic modifications seem to be crucial in aging and longevity processes since they can integrate genetic and environmental factors.

The present study has identified fifteen CpG sites that whose methylation levels showed statistical differences between younger and older subjects that exhibited at least one metabolic alteration and were significantly associated with longevity-regulating pathways. As expected, in our population study, older subjects were more prone to develop metabolic alterations, where no gender differences were found. According to the enrichment pathway analysis and linear regression models, a total of six CpG sites (located at the genes *MTOR*, *ADCY6*, *IGFR1*, *ULK1*, *CREB5* and *RELA*) were common to individuals that exhibited at least one metabolic alteration related to aging. One of these CpG sites (cg08862778) is located at *MTOR* gene, which codifies the mammalian target of rapamycin (mTOR), a serine/threonine protein kinase that can form two complexes: mTORC1 and mTORC2 [34]. This protein can regulate protein translation, protein homeostasis and cellular growth due to its capacity as energy sensor [34]. mTOR can be negatively regulated by rapamycin and caloric restriction, and inhibition of mTORC1 activity is known to increase lifespan in yeast, nematodes, flies and mice [35]. In humans, no associations have been found in SNPs for mTOR complex components in cases of extreme longevity [36], however, mTOR has been shown to be deregulated in several aging-related pathologies such as obesity, diabetes, cardiovascular disease and cancer [34]. Higher levels of mTOR and a chronic activation of mTORC1 in tissues from obese mice and humans appears to play a key role in the development of insulin resistance and type 2 diabetes, which is supported by the results of treatments with metformin, which is known to negatively regulate the action of mTOR [37]. Our results show that cg08862778 (*MTOR*) is hypomethylated in older subjects and also, in those subjects who exhibit abdominal obesity, hypercholesterolemia, insulin-resistance and/or metabolic syndrome. This CpG site is located in a genomic region known as “Transcription start sites” (TSS200) that belong to the promoter region [38], and could be associated with an upregulation of *MTOR*, but this need to be demonstrated since there is evidence for cases where hypomethylation is associated with gene upregulation in autoimmune diseases [39,40] and early stages of tumorigenesis.

A second CpG site hypomethylated in older subjects (cg11658986) is located in the first exon of *ADCY6* gene. *ADCY6* encodes adenylyl cyclase 6, a key protein in the synthesis of cyclic AMP from ATP [41]. ADCY family encodes at least 9 closely related isoforms (1-9) and shares a large sequence homology [42] with functions on learning and memory, olfaction and cardiac contractility [43], but up to date, there is no evidence in metabolism. Nevertheless, genome-wide association studies have shown that *ADCY3* polymorphisms (rs2033655 and rs1968482) are associated with obesity [44] and other SNPs are involved in proximal gene regulation through changes in DNA methylation [45].

Another CpG hypomethylated site corresponds to cg01284192, located in the body of *IGF1R* gene. This gene encodes a receptor that binds insulin-like growth factor with high affinity and plays a key role in cell growth and survival control. It is overexpressed in several types of cancer and has been implicated in the transformation into malignant cells and cell survival promotion [46]. Although it should be necessary to measure IGF1R expression levels, *IGF1R* gene has been reported to be hypomethylated in placentas exposed to maternal impaired glucose tolerance, which suggests its potential implication in fetal programming [47]. Additionally, heterozygous loss-of-function mutations in the *IGF1R* were found to be enriched in the cohort of Ashkenazi Jewish centenarians compared to controls [48]. All these sets of evidences suggest *IGF1R* as a possible candidate gene in aging-related processes [48].

Our results show that three of six CpG sites differentially methylated (in *RELA*, *CREB5* and *ULK1* genes) also correlated with age. Methylation levels for cg08128650, located in the body of the *RELA* gene, are associated negatively with age. *RELA* encodes a transcription factor known as p65, a subunit of NF-κB [49]. This complex is involved in cellular response against several stimuli such as stress, cytokines, UV radiation, oxidized LDL and some bacterial or viral antigens [50]. Methylation at RelA subunit can modulate DNA binding and transcriptional activity, and a deregulation of NFkB, mainly a constitutively activation, is associated with inflammatory and tumorigenesis development [51,52]. The hypomethylation of *RELA* that we have found supports its role; nevertheless, DNA methylation within the gene body is less understood and requires further analysis.

A second differentially methylated site is cg11301281, which exhibits a negative association with age and is located in the 5´ UTR of *CREB5* gene. *CREB5* encodes cyclic AMP-responsive element binding protein 5, a key factor in cell growth, proliferation, differentiation and cell cycle control [53]. Up-regulation of CREB5 is associated with metastatic process [54], inflammatory response genes and modulation of immune responses [55–57]. It is well known that aging is associated with an inflammatory state that contributes to the pathogenesis of several diseases. Transcriptomic and epigenetic analyses in nonagenarian men revealed that *CREB5* methylation was related to inflammatory response genes in a gender-specific manner [58]. In comparison with our results, we did not find gender differences, but our age spectrum is wider and cannot be compared with a nonagenarian population. A third CpG (cg07199984, located in the TSS200 region of *ULK1* gene) was hypermethylated in older individuals. *ULK1* encodes Unc-51 Like Autophagy Activating Kinase 1 (ULK1), a serine/threonine-protein kinase involved in autophagy in response to starvation [59]. Autophagy is a cellular degradation and recycling process that is highly conserved in all eukaryotes [55] and is associated with an extension in lifespan [60]. Under conditions of amino acid starvation or mTOR inhibition, ULK1 phosphorylates Beclin-1 to let a complete induction of autophagy [61,62]. Autophagy has a key role in lipid homeostasis after lipid mobilization lowering their potential toxicity [63,64] and it is well stablished that autophagy dysfunction is linked to aging-related pathologies such as Alzheimer´s disease and type 2 diabetes [65]. Our results show a hypermethylation that could disrupt transcription factor binding to the gene, suggesting a potential silencing of *ULK1* transcription and inhibition of autophagy [66]. This result is related also to the hypomethylation reported for cg08862778 at *MTOR* gene, which suggests mTOR activation which can promote lower levels of autophagy [67,68].

In an attempt to evaluate age-specific CpGs, we perform a similar analysis taking into account only old subjects, but we did not identify the same results in CpGs associated with BMI. We thought that differences between analyzing the whole population and subjects divided by age, are due to different exposures along the lifetime and due to this is not a follow-up study. However, in the young population we can identify a few CpGs in comparison with old subjects, which suggests that aging is driving methylation changes. When older subjects are analyzed as a separate group, we identified two genes that are differentially methylated in metabolic disturbances, and also are associated with BMI in the whole population, suggesting a potential role in metabolic disturbances. *IRS1* belongs to the Insulin receptor substrate 1, and DNA promotor methylation and expression in human adipose tissue are related to fat distribution and metabolic traits [69]. *EHMT1* gene codifies a histone methyltransferase to promote transcriptional repression; a key process in gene regulation associated to aging, and also is associated to the control of brown adipose cell fate and thermogenesis [70,71].

The epigenome, including DNA methylation signatures, undergoes a notable changes during the lifetime and undoubtedly can influence the aging process [19]. For this reason, epigenetic clocks are promising biomarkers of aging [72]. Recently, it has been developed a DNAm age estimator based on 391 CpG sites [73]. This epigenetic clock allows to track the dynamic aging of cells and can be used as a quantitative biomarker of chronological age [73]. In our work, we designed a z-score including individual z-scores from 25 CpG sites, showing that older subjects presented hypomethylated in CpG sites associated with longevity-regulating pathways, which supports the genomic hypomethylation hypothesis, but age-related changes in DNA methylation occurs in specific regions or at specific sites in the genome [74]. Z-scores can be useful predictors because they normalize variables and eliminate a number of the sources of variance in raw values [75]. These results could be related to epigenetic clocks that allow to calculate the acceleration of aging and predict the risk of age-related diseases, cognitive and physical decline, among others [76,77]. One of the remaining challenges in DNA methylation is identify causal pathways that contribute to functional changes and unravel potential mechanisms, but it is important to address the limitations of this type of research. DNA methylation microarrays were performed in buffy coat, which is a mixture of circulating white cells. To circumvent this problem, the results have been corrected using the Houseman procedure [78]. On the other hand, gene expression (mRNA) levels should be determined in order to properly evaluate the impact of the epigenetic changes. To elucidate the mechanisms, it is key to contextualize gene expression with its impact in disease development [79] and taking into consideration other mechanisms (non-coding RNAs, chromatin reorganization, histone modifications) that can also modulate the aging process. Microarrays have the limitation of including only a small amount of the CpGs present in each gene, but it is an affordable technique that can detect individual CpGs in 99% of known genes including 5`UTR, 3`UTR, coding regions and island shores, moreover, Infinium Human Methylation 450K (Illumina) platforms results has a good correlate when has been analyzed by pyrosequencing [80–83]. For this reason, it could be interesting to analyze the methylation status of the whole sequence of the specific genes of interest through specific techniques such as PCR and sequencing, bead array, pyrosequencing, methylation specific PCR [84]) to confirm the differential patterns. In an attempt to compare our results with those from existing databases available in GEO, a public repository of databases, we identified some common patterns in metabolic tissues, particularly in the liver of obese and non-obese individuals, which in turn reinforces our findings and suggests that the CpGs identified in the present study could be used as potential biomarkers.

In the case of aging-longevity methylation studies, it is important evaluate lifestyle and dietary factors, such as methyl donor intake, and study folate metabolism in these subjects to obtain accurate conclusions [85]. Nevertheless, if DNA methylation assays are robust in blood samples, it is feasible the development of epigenetic biomarkers as a method for diagnosis and personalized treatments through a noninvasive approach [86], especially considering the fact that during aging the genome shift towards to a global hypomethylation and hypermethylation in specific sites. In brief, this study has identified several CpG sites that are differentially methylated between younger and older adults and are associated with metabolic dysfunctions.

In summary, our data support that differentially methylated sites could be involved in the promotion of a pro-aging phenotype in those subjects older than 45 years old and in those who exhibit metabolic disturbances. Beyond the limitations of this research and after further validation, these CpG sites could be used as markers of premature aging, especially in the context of obesity and related metabolic diseases. This set of methylation-based biomarkers could be useful in the implementation of new personalized preventing strategies and in the measurement of outcomes to demonstrate the effectiveness of treatments targeting longevity pathways.

## MATERIALS AND METHODS

### Subjects

DNA methylation profiles were analyzed in samples from the Methyl Epigenome Network Association (MENA) project. MENA project comprises 474 adults from previous cohorts analyzed as previously described [87]. Investigation has been conducted in accordance with the ethical standards and according to the Declaration of Helsinki and according to national and international guidelines and has been approved by the authors' institutional review board.

### Participant characteristics

All subjects were categorized according to data obtained from previous cohort databases, including anthropometric measures, blood pressure and metabolic profiles. Body mass index (BMI) was calculated as weight in kilograms divided by the square of height in meters (kg/m^2^) and divided in three categories: normal weight (BMI 18.5–24.9 kg/m^2^), overweight (BMI 25.0–29.9 kg/m^2^) and obesity (BMI ≥ 30 kg/m^2^). Total fat mass was measured by dual-energy X-ray absorptiometry (DXA). Hypercholesterolemia was considered with 200 mg/dl. Homeostatic model assessment-insulin resistance (HOMA-IR) index was calculated as fasting insulin (μIU/ml) × fasting glucose (mmol/ml)/22.5. An HOMA-IR index <2.5 was considered normal and index ≥2.5 was considered insulin resistant (IR). Subjects were considered to have metabolic syndrome (MetS) when having three of the following five listed criteria: large waist circumference (WC) (>102 cm for, men >88 cm for women), reduced high-density lipoprotein (HDL) cholesterol (<40 mg/dl for men, <50 mg/dl for women), high triglyceride level (<150 mg/dl), increased blood pressure (HT) (systolic pressure ≥ 90 mmHg, diastolic pressure ≥ 140 mmHg) and elevated fasting glucose (≥ 100 mg/dl) [88].

### DNA methylation analyses

Blood samples were collected after 8-12 hours fasting on EDTA-containing tubes. White blood cells were isolated from whole blood through differential centrifugation (3500 rpm, 4 °C, 15 min) and frozen at -80ºC. Genomic DNA was extracted from leukocytes with the Master Pure kit (Epicenter, Madison, WI). DNA integrity was further assessed with the Pico Green dsDNA Quantitation Reagent (Invitrogen, Carlsbad, CA). To convert cytosine into uracil, high quality DNA samples (500 ng) were treated with bisulfite using EZ-96 DNA Methylation kit (Zymo Research, Irvine, CA) following manufacturer´s instructions.

Subsequently, DNA methylation was analyzed through microarrays assays using the Infinium Human Methylation 450 K bead chip technology (Illumina, San Diego, CA). Probes intensities data were obtained from 450 k Chip Analysis Methylation Pipeline package [89] for R software (version 1.11.0). Afterwards, probes were filtered according these criteria: p-values >0.01 in at least one sample, beadcounts < 3 in minimum 5% of samples, presence of single nucleotide polymorphisms, alignment to multiple locations or located on X and Y chromosomes. Subset-quantile Within Array Normalization method (SWAN) was used to improves the results obtained from Illumina platform reducing technical variation within and between arrays [90] and ComBat method was applied to adjust for batch effects and eliminate technical variation [91]. Furthermore, DNA Methylation was corrected by cell composition (granulocytes, monocytes, B cells, CD8+ cytotoxic cells, CD4+ helper T cells and natural killer cells) using the Houseman algorithm [78]. DNA methylation for each CpG site was represented by beta values ranging from 0 to 1, corresponding to fully unmethylated and fully methylated, respectively.

### GEO database analysis

In an attempt to validate our results, we decided to perform a search of identified CpG sites in Gene Expression Omnibus (GEO), a public repository. To obtain comparable results, GEO datasets were filtered according these criteria: DNAm arrays performed in Infinium Human Methylation 450K platform with normalized beta values and the presence of metabolic phenotype among groups. To avoid problems when databases are mixed due to differential treatments or experimental conditions, we evaluate specific CpG sites in individual datasets to address hypo or hypermethylation using mean values (p<0.05) and then compare with our results in terms of methylation patterns. Datasets used for this analysis were: GSE76285 (PBMC), GSE65057 (liver tissue), GSE67024 (subcutaneous fat) and GSE54776 (visceral fat) (Supplementary Table 2).

### Pathway analyses

The Kyoto Encyclopedia of Genes and Genomes (KEGG) pathway database (https://www.genome.jp/kegg/) was used to identify genes implicated in “Longevity-regulating pathways” (map04211) which includes insulin, adiponectin, SIRT1, PI3K-Akt, mTOR, AMPK, autophagy, p53, NF-κB, CREB, PGC-1α and FOXO signaling pathways. Additionally, the statistical analysis of longevity-regulating pathways was performed using the Pathway Data Integration Portal (pathDIP), (http://ophid.utoronto.ca/pathDIP/) as previously described [92]. Extended pathway associations with a confidence level for predicted associations of 0.99 were selected, and the p-value for KEGG pathway source was reported.

### Statistical analyses

Quantitative and qualitative variables were expressed as means ± standard deviations (SD) or standard error of the mean (SEM). After pre-processing of the methylation data, linear regression adjusted for potential confounding factors (age, sex, study cohorts and DNA methylation chips) was carried out with the LIMMA package for R software (v. 3.3.2). A False Discovery Rate (FDR) cut-off of 0.05 and LIMMA B-statistics values above 0 in the outcome-related analyses were used as statistically significant thresholds. The LIMMA B-statistic is the log-odds of differential methylation, where B-values > 0 implies that the CpG is more likely to be differentially methylated than to not be differentially methylated [90]. This cut-off value (B > 0) gives a feasible balance between false positives and false negatives. FDR values (p< 0.0001) were used to select those CpGs whose methylation levels strongly correlated with BMI. The standard scores (z-score) were calculated as the sum of individual z-score for each CpG site (beta values) using this formula: z = x – μ / σ, where x (sample value), μ (sample mean) and σ (sample standard deviation). Moreover, adjusted linear regression analyses were performed to evaluate correlations between z-score and age, adjusted by BMI and methylation levels at genes of the longevity-regulating pathways and four metabolic traits: Waist circumference, hypercholesterolemia, HOMA-index and metabolic syndrome. Linear regressions results were expressed as raw regression (r) and r-squared (r^2^) coefficients. Data was considered significant at p values <0.05. Statistical analyses were performed using IBM SPSS software, version 20 (IBM Inc., Armonk, NY, USA) and plots were created using GraphPad Prism® software, version 6.0C (La Jolla, CA, USA). Accession number in Gene Expression Omnibus [GEO] database: GSE115278.

## Supplementary Material

Supplementary Figures

Supplementary Tables
