# Supplementary material for: DNA methylation in genes of longevity-regulating pathways: association with obesity and metabolic complications
Source: Aging (Albany NY). 2019 Mar 29;11(6):1874–99. doi: 10.18632/aging.101882 (PMC6461164; doi:10.18632/aging.101882)
Supplement: Supplementary Figures [file aging-11-101882-s001.pdf]

SUPPLEMENTARY FIGURES

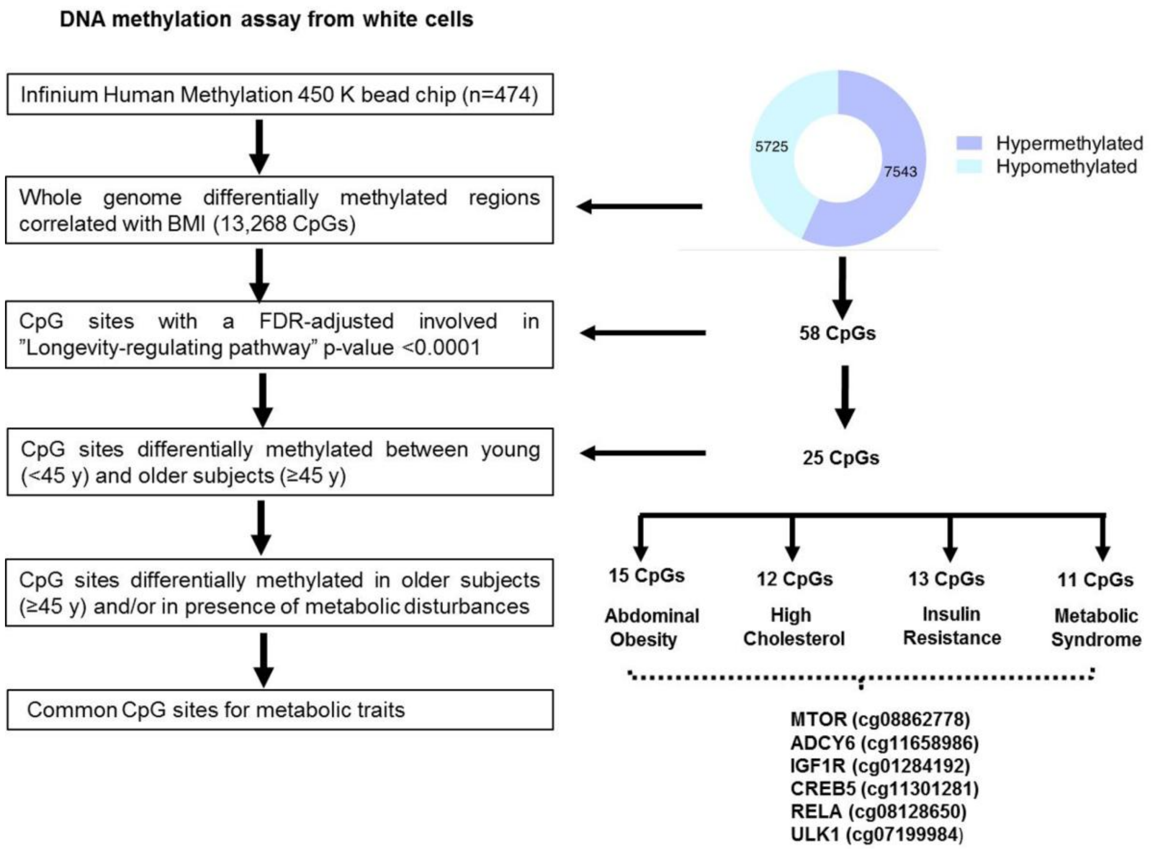

Supplementary Figure S1. Scheme of CpG selection process for the analyses.

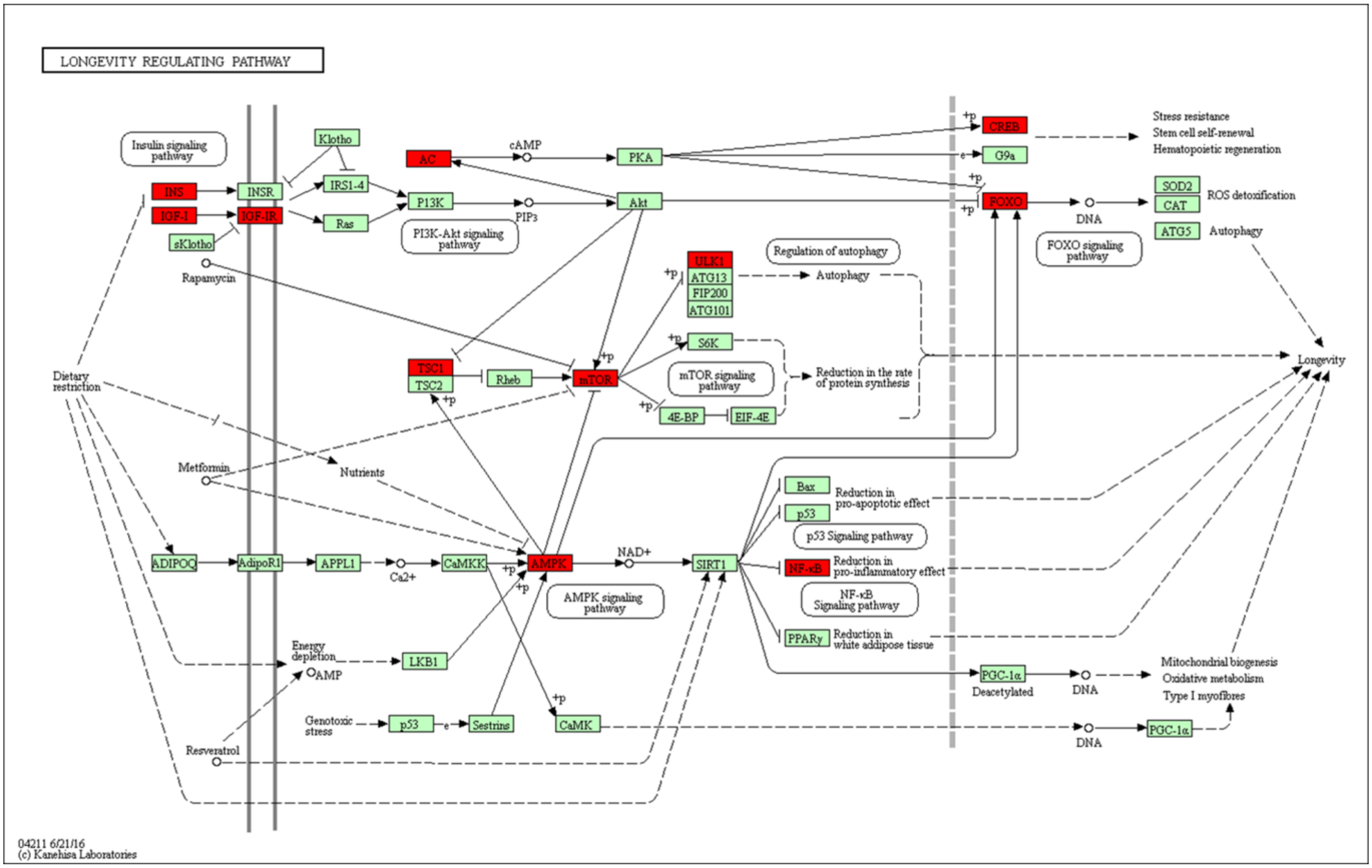

Supplementary Figure S2. Longevity Regulating Pathways genes. Red boxes correspond to those CpG sites differentially methylated associated to older subjects and the presence of metabolic disturbances. Diagram modified from KEGG Database.
