# Supplementary material for: DNA methylation in genes of longevity-regulating pathways: association with obesity and metabolic complications
Source: Aging (Albany NY). 2019 Mar 29;11(6):1874–99. doi: 10.18632/aging.101882 (PMC6461164; doi:10.18632/aging.101882)
Supplement: Supplementary Tables [file aging-11-101882-s002.pdf]

SUPPLEMENTARY TABLES

Supplementary Table 1. CpG sites associated with BMI residing in longevity genes (FDR<0.0001) in the whole population.

	CpG_ID	P.Value	adj.P.Val	Gene
1	cg21511036	8,96E-15	5,01E-11	*IRS1*
2	cg08862778	1,83E-13	3,37E-10	*MTOR*
3	cg11322849	1,57E-10	5,14E-08	*INS*
4	cg20406576	4,20E-10	1,07E-07	*PRKAG2*
5	cg13551841	3,44E-09	5,12E-07	*EHMT1*
6	cg10136773	1,07E-08	1,17E-06	*SESN3*
7	cg07199894	1,38E-08	1,42E-06	*ULK1*
8	cg01749142	1,49E-08	1,51E-06	*AKT1*
9	cg11658986	1,58E-08	1,57E-06	*ADCY6*
10	cg21195984	1,91E-08	1,82E-06	*EHMT1*
11	cg15283498	2,18E-08	2,01E-06	*FOXO3*
12	cg14862787	4,15E-08	3,20E-06	*CREB5*
13	cg05792022	4,78E-08	3,55E-06	*FOXO1*
14	cg15973818	5,65E-08	4,00E-06	*RB1CC1*
15	cg22048274	6,24E-08	4,28E-06	*EHMT2*
16	cg13810766	6,83E-08	4,57E-06	*PRKAG2*
17	cg23709782	1,27E-07	7,22E-06	*IGF1R*
18	cg17848496	1,39E-07	7,72E-06	*IRS1*
19	cg04149773	1,39E-07	7,72E-06	*ADCY6*
20	cg02352203	1,42E-07	7,83E-06	*RPTOR*
21	cg14113970	2,16E-07	1,05E-05	*CAMK4*
22	cg14129040	2,30E-07	1,10E-05	*CREB1*
23	cg14844401	2,31E-07	1,11E-05	*ADCY5*
24	cg03813033	3,01E-07	1,33E-05	*AKT1S1*
25	cg07012178	3,07E-07	1,35E-05	*PRKAG2*
26	cg14267811	3,24E-07	1,41E-05	*TSC1*
27	cg01284192	4,12E-07	1,68E-05	*IGF1R*
28	cg24061580	4,71E-07	1,84E-05	*PRKAG2*
29	cg01091261	5,50E-07	2,06E-05	*ADCY9*

	CpG_ID	P.Value	adj.P.Val	Gene
30	cg06223834	6,22E-07	2,25E-05	*ADCY9*
31	cg18028483	6,40E-07	2,29E-05	*SESN1*
32	cg12416929	7,54E-07	2,58E-05	*FOXO3*
33	cg12566890	8,43E-07	2,80E-05	*ADCY2*
34	cg13574337	9,35E-07	3,01E-05	*ADCY9*
35	cg04832916	1,00E-06	3,18E-05	*CAMKK2*
36	cg13796676	1,12E-06	3,43E-05	*SIRT1*
37	cg06772578	1,30E-06	3,83E-05	*PPARGC1A*
38	cg19503731	1,31E-06	3,85E-05	*AKT3*
39	cg10421188	1,36E-06	3,95E-05	*CREB5*
40	cg13613346	1,49E-06	4,22E-05	*EHMT2*
41	cg18758433	1,52E-06	4,28E-05	*RPTOR*
42	cg13154908	1,58E-06	4,39E-05	*PIK3CA*
43	cg24937356	1,96E-06	5,13E-05	*PRKAA2*
44	cg18237616	2,02E-06	5,25E-05	*RHEB*
45	cg14323456	2,06E-06	5,33E-05	*RHEB*
46	cg01781374	2,12E-06	5,44E-05	*CAMK4*
47	cg19292222	2,27E-06	5,70E-05	*RPTOR*
48	cg11301281	2,48E-06	6,08E-05	*CREB5*
49	cg02823066	2,54E-06	6,18E-05	*IGF1*
50	cg20300093	2,80E-06	6,63E-05	*ADCY5*
51	cg14077232	2,80E-06	6,63E-05	*EHMT1*
52	cg04932465	3,35E-06	7,52E-05	*CAMKK2*
53	cg08128650	3,40E-06	7,61E-05	*RELA*
54	cg08315825	3,61E-06	7,96E-05	*IGF1R*
55	cg19418273	3,89E-06	8,40E-05	*CREB3L2*
56	cg17324121	3,93E-06	8,44E-05	*IRS2*
57	cg00210002	4,12E-06	8,76E-05	*EHMT2*
58	cg14072989	4,51E-06	9,33E-05	*RPS6KB1*

CpG_ID: CpG loci identifier in Illumina platform
Adj. P. Val: Adjusted P value

Supplementary Table S2. GEO datasets from metabolic studies using DNA methylation arrays with Infinium Human Methylation 450K (Illumina platform).

Geo Accession	Sample Tissue	Sample Size	Gender	Age (y)	BMI (kg/m²)	Study Title
GSE76285	PBMC	80	Women	36.4 ± 6.3 (Insulin Sensitive) 35.7 ± 5.7 (Insulin Resistant)	>35 kg/m² (with comorbidities) or >40 kg/m²	The epigenetic signature of systemic insulin resistance in obese women. PMID: 27535281
GSE65057	Liver tissue	15	Men	40.5 ± 4.1 (Non Obese) 41.6 ± 4.1 (Obese)	26.1 ± 2.0 kg/m² (Non Obese) 38.9 ± 2.1 kg/m² (Obese)	Altered DNA methylation of glycolytic and lipogenic genes in liver from obese and type 2 diabetic patients. PMID: 26977391
GSE67024	Subcutaneous fat	29	Women	45 ±11 (Non Obese) 46 ±11 (Obese)	25.2 ± 2.5 kg/m² (Non Obese) 41.3 ± 4.4 kg/m² (Obese)	The epigenetic signature of subcutaneous fat cells is linked to altered expression of genes implicated in lipid metabolism in obese women. PMID: 26351548
GSE54776	Visceral fat	14	Men	33.6 ± 5.8 (Met Syndrome -) 42.4 ± 10.9 (Met Syndrome +)	50.7 ± 3.5 (Met Syn -) 55.8 ± 8.4 (Met Syn +)	Gene methylation profiles in visceral adipose tissue of obese mens with or without metabolic syndrome. PMID: 24495915

BMI: Body Mass Index
PBMC: Peripheral Blood Mononuclear Cells
